# New Model of Disuse-Induced Bone Density Loss in Horses †

**DOI:** 10.3390/ani15213137

**Published:** 2025-10-29

**Authors:** Lisa Micheau, Fabrice Audigié, Claire Moiroud, Sandrine Jacquet

**Affiliations:** 1 Ecole Nationale Vétérinaire d’Alfort, ACAP3, F-14430 Goustranville, France; fabrice.audigie@vet-alfort.fr (F.A.); claire.moiroud@vet-alfort.fr (C.M.); sandrine.jacquet@vet-alfort.fr (S.J.); 2 Ecole Nationale Vétérinaire d’Alfort, F-94700 Maisons-Alfort, France

**Keywords:** equine, bone density, demineralization, CTX-I, equine model

## Abstract

Disuse-induced bone loss during stall confinement and immobilization is a major concern in horses because it impairs recovery and increases susceptibility to further injury. Experimental models are needed to evaluate therapeutic options, but most available equine models rely on cast immobilization, which is technically demanding and may be associated with complications. This study aimed to assess a simpler and less restrictive model to induce a quantifiable decrease in bone density in horses. Six French Standardbred horses underwent eight weeks of stall confinement, with a wooden wedge fitted to one front foot to elevate the heels during the last four weeks. Bone density was assessed using computed tomography (CT) examinations of both forelimbs performed at the beginning (M0) and after the confinement period (M2). Serum markers of bone metabolism (CTX-I, osteocalcin, bone-specific alkaline phosphatase, and hydroxyproline) were analyzed monthly from baseline to 2 months post confinement. Statistical analysis used Wilcoxon signed-rank tests and mixed models as appropriate. Computed tomography revealed a significant decrease in bone density after confinement (*p* < 0.05), more pronounced distally in the wedge limb. CTX-I levels varied with physical activity. This model provides a practical and reproducible alternative to cast immobilization for inducing equine bone demineralization.

## Simple Summary

Bone loss due to reduced mechanical loading is a well-recognized phenomenon in both human and veterinary medicine. Experimental models of disuse-induced bone density in horses typically rely on cast immobilization, which can be associated with complications. In this study, we investigated an alternative approach based on stall confinement combined with unilateral heel elevation to induce skeletal unloading in the forelimb. Using computed tomography (CT) imaging, we observed significant bone loss over a two-month period, particularly in the limb with elevated heels. These findings support the use of this model as a practical alternative for developing new pharmacological targets in horses.

## 1. Introduction

Immobilization is often used in the early stages of recovery to promote the healing of musculoskeletal injuries. However, mechanical loading is a key regulator of bone mass and density [1], and a reduction in applied forces shifts the balance toward bone resorption rather than formation [2,3,4]. This disuse-induced bone loss, commonly termed ’disuse-osteoporosis’, has been documented in several species [5], with detrimental effects on structural and biomechanical properties, thus increasing fracture risk and prolonging recovery. In horses, bone demineralization during periods of stall confinement is a major clinical issue, as it compromises healing and increases susceptibility to secondary injuries [6,7,8,9]. To better understand and prevent disuse-induced bone loss, reliable models are required. Such models are also essential for the development and evaluation of therapeutic strategies that aim to limit bone resorption. Several animal models of disuse-induced bone loss have been widely used, mainly in rodents [5,10], but reliable equine-specific models remain essential, as pharmacological strategies must ultimately be tested in the target species. In horses, bone loss associated with stall confinement has already been described [7]. Stall confinement combined with cast immobilization is the most commonly used model to induce disuse-induced bone density loss, and it has enabled the evaluation of therapies such as bisphosphonates [11]. However, this approach requires the skilled application of the cast in horses [12,13] and can result in complications such as soft tissue injuries, tendon damage, or even irreversible musculoskeletal changes that lead to discomfort and pain, as previously described in the literature [13,14,15]. In a multicenter retrospective case study of 398 horses treated with a half-limb or full-limb cast, sores were the most commonly reported lesion (45%) [13]. Although cast models remain scientifically valuable, their practical demands, possible complications, and potential welfare concerns highlight the need for an alternative approach. The purpose of this experimental study was therefore to describe and evaluate a simpler, reproducible, and less invasive model of disuse-induced bone density loss in horses. We hypothesized that 2 months of stall confinement, combined with elevated heels on a single forelimb during the last month, would induce significant and quantifiable bone changes detectable by computed tomography (CT) and would be associated with modifications in biochemical markers of bone turnover.

## 2. Materials and Methods

All experimental procedures were approved by the National Ethics Committee on Animal experimentation: ComEth Anses/ENVA/UPEC (with the approval number APAFIS #34943-2022012118055143, 19 February 2022).

### 2.1. Horses

Six French standardbred horses [5 females, 1 gelding, median age 3 years (interquartile range: 2–5), and median weight 447 kg (range 407–500)] were used. The horses belonged to the experimental herd of the National Veterinary School of Alfort (ENVA). Each horse was vaccinated and dewormed. The horses were kept in the same pasture for 4 weeks before starting the protocol, to ensure uniformity in their environmental conditions, primarily to minimize the effect of diet, activity, and daylight exposure. During the stall confinement period, the horses had free access to low-nutrient hay and were fed one concentrate meal per day to meet their daily needs.

### 2.2. Experimental Design

The day before the start of the study, all horses underwent a complete clinical examination including auscultation, complete blood analysis, and a physical examination with palpation and joint mobilization performed by an experienced clinician. A dynamic examination was also performed, consisting of gait evaluation at the walk and trot on a straight line, with flexion tests performed on all limbs. A radiographic examination of both distal forelimbs was also performed to rule out any significant bone and joint lesions.

**Figure 1:**
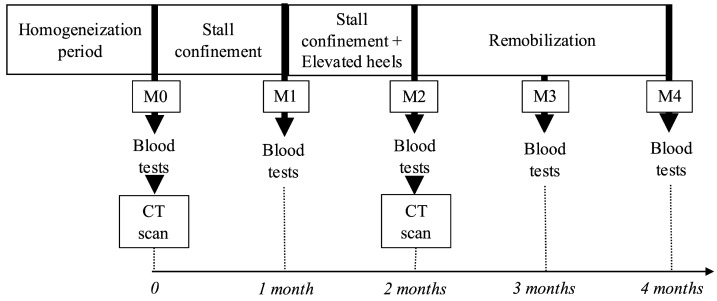
Experimental timeline of the disuse-induced bone loss model in horses based on stall confinement and elevation of heel on one forelimb. The figure illustrates the study design over a 4-month period, including the main experimental phases. Computed tomography (CT) scans and blood samples collected at key time points as indicated.

At the start of the study (M0), the horses were confined to stalls for a period of 2 months (M0 to M2) (Figure 1), housed in 3 m × 2 m box stalls, and evaluated daily by a veterinarian. Daily monitoring included physical examination; auscultation to monitor heart rate; and the evaluation of attitude, appetite, and level of comfort. During the entire confinement period, horses had free access to water and hay and could lie down and rise freely within the stalls, even when equipped with the wedge.

**Figure 2:**
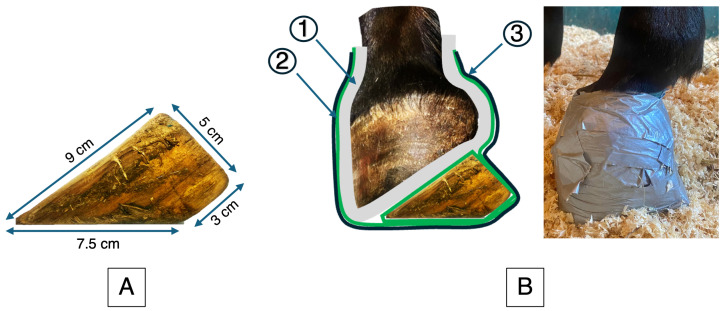
Wooden wedge and its application under the hoof. (**A**) Dimensions of the wooden wedge used in this study (length = 7.5 cm; height = 5 cm; base = 3 cm; hypotenuse = 9 cm; and approximate angle = 33°). (**B**) Application of the wedge under the hoof. (1) Cotton padding to protect the foot, (2) cohesive bandage for fixation, and (3) adhesive tape for reinforcement and waterproof.

For the last month of stall confinement, a 5 cm wooden wedge was applied by a veterinarian to one randomly selected front foot (Figure 2) and secured with a hoof bandage. The wedge was checked daily for proper fitting and replaced twice a week. At each change, the veterinarian carefully examined the skin and assessed for signs of pain. Stall confinement was then followed by two months of remobilization (M2 to M4), during which the horses were returned to pasture.

### 2.3. Computed Tomography

All horses underwent computed tomographic (CT) examination of both forelimbs under general anesthesia at the beginning of (M0) and after the confinement period (M2). The horses were positioned in lateral recumbency on the side of the limb fitted with the wedge during confinement. Two acquisitions were systematically performed, one for each forelimb positioned alone within the gantry. Particular care was taken to align the dorsal cortices of the metacarpal bone and phalanges. Scans were obtained from the carpus to the distal extremity of the hoof (Toshiba Aquilion LB; Canon Medical Systems, Otawara, Japan; 120 kV, 250 mA, small focal spot, 0.5 s rotation, FOV 119.7 mm, 512 × 512 matrix, 0.5 mm slice thickness, 700 mm scan length, and acquisition time 65 s). The transverse images were reconstructed with a bone filter at 1 mm slice thickness and 20% overlap.

Each CT acquisition series was processed in multiplanar reconstruction (MPR) mode to generate transverse slices perpendicular to the long axis of the third metacarpal bone (McIII). Using the MPR tool, each acquisition series was recalculated in two standardized DICOM series of 256 transverse slices per limb: (i) the “McP” series, from the carpometacarpal joint to the distal part of the condyle of the proximal phalanx, and (ii) the “Dist” series, from the apex of the proximal sesamoid bones to the distal extremity of the distal phalanx. These DICOM recalculated series were analyzed using IMAGE J software, version 1.53t (National Institutes of Health, Bethesda, MD, USA) (https://imagej.nih.gov/ij/index.html (accessed on 24 August 2022). Each slice of the series was segmented using a fixed threshold of 400 HU. Pixels above this value were selected, and their HU values were summed to obtain the total HU value of the slice. For each series, the total HU value of the 256 slices was added to calculate the total HU value of the series. This total HU value of the series was used to evaluate the total bone density of the bones present in the images of the series “McP” and “Dist”.

To control for potential scanner drift in the HU values, a bone phantom from the same anatomical region was scanned at each session using the same CT acquisition parameters.

### 2.4. Measurement of Serum Concentrations of Biochemical Markers

In this study, biomarkers previously applied in equine studies were evaluated to assess bone formation and resorption [9,11,14,16,17]. Blood samples were taken at baseline (M0), mid-confinement (M1), and at the end of confinement (M2), as well as after one (M3) and two months (M4) of remobilization at 9:00 a.m. Figure 1. Serum and heparinized samples were refrigerated and analyzed the same day at an external laboratory facility (GIP Labeo, Saint-Contest, France). Bone synthesis was assessed by measuring osteocalcin (OC) and bone-specific alkaline phosphatase (B-ALP). Osteocalcin concentrations were measured using an ELISA kit (N-MID® Osteocalcin ELISA; Immunodiagnostic Systems Ltd., Boldon Colliery, UK). Bone-specific alkaline phosphatase was quantified by agarose gel electrophoresis using the HYDRAGEL 154 ISO-PAL kit (PN 4132, Sebia, Lisses, France). Electrophoresis was performed on a semiautomated Hydrasys system and visualized using the Hydrasys Focusing scanner (Sebia, Lisses, France). Bone resorption was assessed by measuring cross-linked C-telopeptides of type I collagen (CTX-I) and hydroxyproline. CTX-I concentrations were evaluated using an ELISA kit (Serum Crosslaps® (CTX-I) ELISA; Immunodiagnostic Systems Ltd., Boldon Colliery, UK). This kit has also been used in a study evaluating in vitro equine bone resorption [18]. Hydroxyproline concentrations were determined using a custom colorimetric method adapted from Woessner et al. [19] based on oxidation with chloramine T and reaction with p-dimethylaminobenzaldehyde, with absorbance read at 558 nm. All ELISA assays were performed according to the manufacturers instructions. These assays are marketed for use in translational research, including animal models.

### 2.5. Statistical Analysis

Given the experimental nature of this study and the limited sample size, statistical analyzes prioritized within-subject paired comparison and mixed-effects models to identify temporal trends rather than to produce precise population-level estimates. All statistical analyzes were performed in R (version 4.2.2, GUI 1.79 High Sierra build; R Core Team, 2022, Vienna, Austria) using RStudio (version 2023.12.1+402).

Two sets of comparisons were performed to assess changes in bone density. First, for each limb (with and without elevated heels) and for each series (McP and Dist), total HU values at M0 and M2 were compared to evaluate temporal changes. Second, for each series, the intraindividual differences in HU values between M0 and M2 were calculated and compared between the limbs to assess the effect of heel elevation. The normality of the data was assessed using the Shapiro–Wilk test. Due to the small sample size, a paired Wilcoxon signed rank test was used for all comparisons.

To assess changes in biochemical markers (CTX-I, B-ALP, osteocalcin, and hydroxyproline) over time, a mixed linear model was fitted using the lme4 package(version 1.1-31) [20], with time as fixed effect and “horse” as a random intercept. The residuals were tested for normality (Shapiro–Wilk) and homogeneity of variance (Bartlett). For PAL and osteocalcin, which met model assumptions, specific contrasts between time points (M0 vs. M1, M0 vs. M2, and M2 vs. M4) were tested using the multcomp package (version 1.4-25) [21], to allow for multiple comparisons with adjustment. For CTX-I and hydroxyproline, which violated normality assumptions, data were first ranked, and the linear mixed effects model was applied to the ranked values. The same set of predefined contrasts was tested in the ranked models.

For each comparison, the statistical significance was set at p<0.05.

## 3. Results

### 3.1. Clinical Examination

No clinical abnormalities were detected during the complete clinical examination performed in all horses the day before the start of the study. No complications related to stall confinement or wedge application, such as sores or signs of discomfort, were observed throughout the study. On the foot fitted with the wooden wedge, a shortening of the toe was observed at the end of the confinement period. No trimming was performed during confinement, but routine trimming at the end of the period restored the usual hoof conformation. After 2 months of immobilization, none of the horses developed lameness, even in the limb on which the wooden wedge had been applied for one month.

### 3.2. Computed Tomography

**Figure 3:**
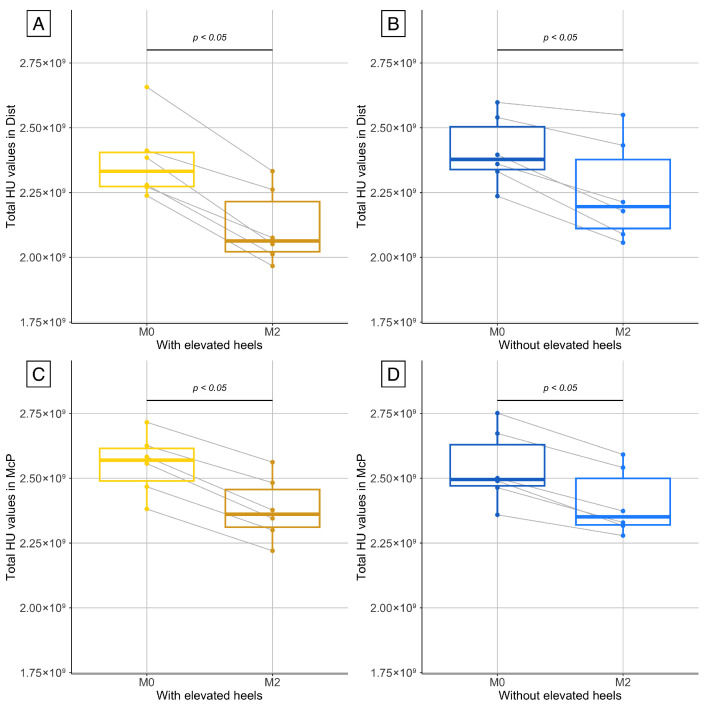
Evolution of total HU values measured by computed tomography (CT) in the Dist (**A**,**B**) and McP series (**C**,**D**) regions of both forelimbs. (**A**,**C**) correspond to the limb fitted with the wooden wedge (elevated heels) during the last month of the confinement period, while (**B**,**D**) correspond to the contralateral limb. Measurements were taken at the beginning of the confinement period (M0, baseline) and after 2 months of stall confinement (M2).

**Figure 4:**
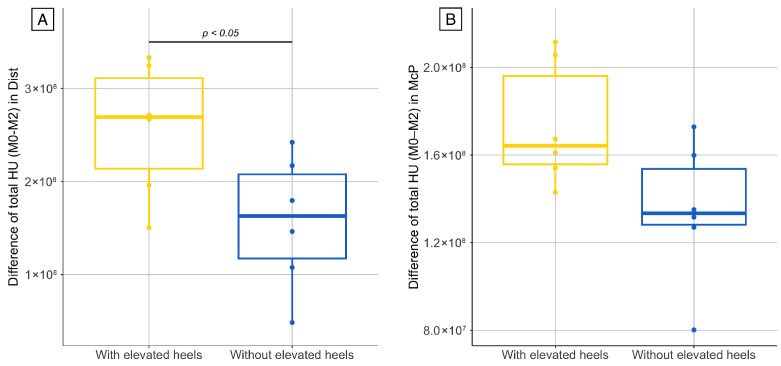
Comparison of bone density loss between forelimbs, based on the difference in total HU values between the beginning (M0, baseline) and the end of the confinement period (M2). Changes are shown for limbs with and without elevated heels during the last month of confinement, in the Dist (**A**) and McP (**B**) series.

The difference in the bone phantom for the total HU value of the series, between M0 and M2, was measured at 1%. Consequently, no correction for HU drift was applied for HU values measured in the horses. A significant decrease in the total HU value of the series was observed between the beginning (M0) and the end of confinement (M2) in the McP and Dist series in the limb with and without elevated heels (Figure 3). This reduction was significantly higher in the limb with elevated heels compared to the contralateral limb in the Dist series, while in McP the difference did not reach statistical significance (Figure 4). The medians and associated *p* values are reported in Table 1 and Table 2.

### 3.3. Serum Concentrations of Biochemical Markers

**Figure 5:**
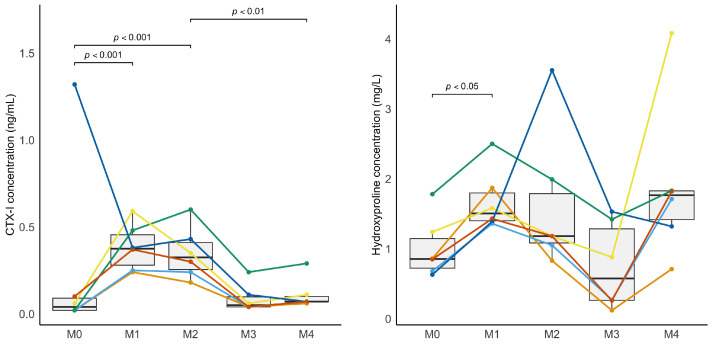
Changes in serum concentrations of C-telopeptides of type I collagen (CTX-I) and hydroxyproline over time. Monthly serum samples were collected from the beginning of the study (M0, baseline) through the confinement period (M1 and M2) and during the remobilization phase (M3 and M4, corresponding to one and two months after return to pasture).

Markers of bone resorption were influenced by the horses’ level of physical activity. Serum CTX-1 concentrations increased significantly during the confinement period (Figure 5), from M0 to M1 and from M0 to M2, and decreased during the remobilization phase, from M2 to M4. At baseline (M0), one horse exhibited a higher serum CTX-I value (1.3 ng/mL) compared to the rest of the group.

Hydroxyproline concentrations increased significantly between M0 and M1, but no significant variations were observed from M0 to M2 or from M2 to M4 (Figure 5). Bone alkaline phosphatase (B-ALP) concentrations decreased significantly during remobilization from M2 to M4. No significant variation in osteocalcin concentrations was observed during this study. Complete descriptive statistics and *p*-values are reported in Supplementary Table S1.

## 4. Discussion

This prospective study on six horses showed that a period of 2 months of stall confinement with a wooden wedge applied to one front foot for the last month was associated with a significant decrease in bone density from the carpus to the toe. A significant loss of total HU value was observed between the beginning and end of the immobilization period in both forelimbs, reflecting a decrease in bone density. The decrease in bone density was more pronounced in the forelimb with elevated heels in its distal part. This supports the hypothesis that the wedge effectively enhances local unloading, thereby amplifying bone resorption in the most mechanically affected areas. This region could therefore represent a relevant area of interest for future investigations, as it appears to be the most responsive to mechanical unloading.

The loss of bone density induced by stall confinement was expected based on the clinical experience of the authors. Several studies have revealed that stall confinement is responsible for bone loss in horses without access to high-speed exercise [7]. In a previous study using radiographic photodensitometry, horses housed in individual stalls and undergoing a progressive conditioning and racing program showed a decrease in radiographic bone aluminum equivalence (RBAE) of the third metacarpal bone on day 28, which persisted throughout the study period of 140 days, even when horses began race training under saddle [22]. In another study, a decrease in bone density was identified in horses during 12 weeks of box stall confinement, although they were walked twice a day on a mechanical walker for 30-min exercise sessions [6]. A wide range of studies have shown that stall confinement adversely affects bone density in horses, leading to disuse osteopenia and increased susceptibility to skeletal injuries [7,8,9].

Our method quantified integrated HU values as a relative index of mineralized tissue. Although HU values are not equivalent to absolute bone mineral density (BMD, in mgHA/cm^3^) without calibration to a density phantom, they are proportional to X-ray attenuation and reflect the degree of mineralization. This approach is consistent with previous work by Stewart et al., who also used the HU map to assess the bone mineralization pattern in the equine limbs before converting the values into absolute units using a calibration phantom [14].

During this study, horses tolerated the wedge well, and no adverse effects, such as sores that are potentially associated with casts, were observed. The wedge was simple to manufacture and apply, providing a practical, effective, and inexpensive option. The use of the wooden wedge allowed the horses to move freely in the stall; they were able to bear weight on the limb but spent more time with the limb in protraction or flexed at rest because of the bulk of the device. The interindividual variability of horse activity in the stall was the main concern during the study. The use of pedometers or camera tracking would have been interesting to assess how much bone density loss is affected by the horse’s level of activity in the stall. As previous studies showed a significant decrease in RBAE of the third metacarpus in the first weeks of stall confinement, even with 30 min of daily walking [7,9]; this suggests that active horses in our study would still have experienced significant bone loss [6]. In addition, unrestricted access to hay may have reduced stress levels, and environmental enrichment (treat-dispensing toy) was also used to prevent boredom. Low-nutrient hay was provided ad libitum to minimize the risk of weight gain during confinement, as increased body weight could have been detrimental to the study.

Variations in biochemical markers of bone turnover were expected in parallel with changes in bone mineralization [22]. In horses, age and breed are known to influence bone turnover markers [16]. The present study minimized these effects by restricting the age range and using a single breed. Serum CTX-I varied significantly over time in response to stall confinement and re-mobilization, indicating that this marker is responsive to changes in bone turnover in horses. These findings are consistent with previous studies, particularly in humans [23], but also in horses, where CTX-I concentrations were shown to correlate with 3D bone resorption volumes [18]. This contrasts with the results of Stewart et al. [14], who reported no consistent differences between time points, although a significant overall decrease was observed from baseline.

In our study, CTX-I serum concentrations increased during confinement and decreased during the remobilization period, closely paralleling the expected resorption pattern. One horse exhibited an isolated high baseline CTX-I value. No specific explanation was found, as all other markers for this horse remained consistent with the group. However, this did not alter the statistical significance of the changes observed over time. Similarly, although hydroxyproline has not been widely used in previous studies, it was also affected in this study at the beginning of the immobilization period (M0 vs. M1). Serum osteocalcin concentration was not significantly affected by the physical activity level in our study, which contrasts with previous studies [9,16,17], and BA-LP only varied significantly during remobilization.

The apparent uncoupling between increased resorption markers (CTX-I, hydroxyproline) and mild or absent variations in bone formation markers (osteocalcin, B-ALP) is consistent with previous reports in humans. Immobilization and weightlessness lead to a rapid sustained increase in CTX-I, whereas bone formation markers such as osteocalcin and B-ALP show little to no change during the early phase of unloading [24]. For hydroxyproline, this marker is not specific to bone and is less commonly used in both human and veterinary medicine, which may explain the partial response to immobilization [16,24].

The duration of stall confinement was chosen based on the results of similar previous studies [5,14,25]. A CT scan could have been performed at M1 to assess whether bone changes suggested by variations in serum CTX-I concentration were associated with an early significant loss of bone density. A recheck CT scan could also have been performed at the end of the remobilization period in M4, to check that bone mineral density was similar to that of M0. The cost of CT scans and repeated general anesthesia were limiting factors. Furthermore, in relation to the primary objective of the study (that is, to assess the effect of the experimental model on the reduction in bone density), these examinations in M1 and M4 were not necessary. Nowadays, CT scanners that allow for standing examinations of the limbs could help address this limitation. Furthermore, a previous study comparing the bone mass of the third metacarpal in horses confined to box stalls with those housed in stalls with exercise or kept on pasture demonstrated that horses regained similar bone mineral content when returning to pasture [7].

The main limitation of the study is the small sample size. As this was an experimental study conducted on a large animal model, the primary objective was to evaluate the feasibility and sensitivity of a novel approach that combined stall confinement with controlled heel elevation using a wooden wedge. The number of horses used reflected a compromise between scientific validity, practical constraints, and adherence to the 3R principles. However, this study highlights a practical alternative to existing models, offering a less restrictive and better-tolerated option for horses while allowing for the controlled study of disuse-induced bone loss. This model could also serve as a relevant experimental platform to test new pharmacological or rehabilitative strategies aimed at counteracting bone loss in convalescent horses.

## 5. Conclusions

This model provides an alternative to cast immobilization for induction of a significant reduction in bone density, which is easier to perform and less restrictive for the horses. This study also highlights the significant loss of bone density related to stall confinement, which has already been reported in various studies.

## Figures and Tables

**Table 1:** Median total HU values at M0 and M2 in Dist and McP series, with and without elevated heels.

Serie	Condition	Time	Median (HU)	IQR	*p*-Value
Dist	With elevated heels	M0	$2.33\times 10^{9}$	$1.40\times 10^{8}$	0.031
		M2	$2.06\times 10^{9}$	$2.49\times 10^{8}$	
	Without elevated heels	M0	$2.38\times 10^{9}$	$2.08\times 10^{8}$	0.031
		M2	$2.20\times 10^{8}$	$3.43\times 10^{8}$	
McP	With elevated heels	M0	$2.57\times 10^{9}$	$1.25\times 10^{8}$	0.031
		M2	$2.36\times 10^{9}$	$1.45\times 10^{8}$	
	Without elevated heels	M0	$2.50\times 10^{9}$	$1.59\times 10^{8}$	0.031
		M2	$2.35\times 10^{9}$	$1.79\times 10^{8}$	

**Table 2:** Median paired difference in total HU values in Dist and McP series, with and without elevated heels.

Serie	Condition	Time	Median (HU)	IQR	*p*-Value
Dist	With elevated heels	M0–M2	$2.69\times 10^{8}$	$1.28\times 10^{8}$	0.031
	Without elevated heels	M0–M2	$1.63\times 10^{8}$	$1.09\times 10^{8}$	
McP	With elevated heels	M0–M2	$1.64\times 10^{8}$	$4.03\times 10^{7}$	0.063
	Without elevated heels	M0–M2	$1.33\times 10^{8}$	$2.55\times 10^{7}$	

## Data Availability

The data presented in this study are available on request from the corresponding author.

## Supplementary Materials

The following supporting information can be downloaded at https://www.mdpi.com/article/10.3390/ani15213137/s1, Supplementary Table S1: Median concentrations and statistical contrasts for serum bone turnover markers (hydroxyproline, CTX-I, B-ALP and osteocalcin) measured at five time points (M0 to M4) during the study.
