# Supplementary material for: New Model of Disuse-Induced Bone Density Loss in Horses
Source: Animals (Basel). 2025 Oct 29;15(21):3137. doi: 10.3390/ani15213137 (PMC12607326; doi:10.3390/ani15213137)
Supplement: Supplementary file 1 [file animals-15-03137-s001.zip › animals-3924838-supplementary.pdf]

Table S1: Median concentrations and statistical contrasts for serum bone turnover markers (hydroxyproline, CTX-I, B-ALP and osteocalcin) measured at five time points (M0 to M4) during the study.

	Median				
**Marker**	**M0**	**M1**	**M2**	**M3**	**M4**
**Hydroxyproline (mg/L)**	0.855	1.505	1.180	0.575	1.765
**CTX-I (ng/mL)**	0.040	0.375	0.325	0.050	0.070
**B-ALP (UI/L)**	45	62.5	55.5	38.5	38.5
**Osteocalcin (ng/mL)**	13.95	14.0	12.60	10.80	6.3

Marker	Contrast	Estimate	p_value	CI_low	CI_high
**Hydroxyproline**	**M0 vs. M1**	-10.667	0.011	-19.398	-1.936
	**M0 vs. M2**	-6.000	0.254	-14.731	2.731
	**M2 vs. M4**	-3.833	0.608	-12.564	4.898
**CTX-I**	**M0 vs. M1**	-14.167	< 0.001	-21.363	-6.971
	**M0 vs. M2**	-12.833	< 0.001	-20.029	-5.637
	**M2 vs. M4**	9.583	0.00467	2.387	16.779
**PAL**	**M0 vs. M1**	-6.333	0.1440	-14.160	1.493
	**M0 vs. M2**	-5.0	0.3110	-12.827	2.827
	**M2 vs. M4**	9.083	0.0172	1.257	16.91
**Osteocalcin**	**M0 vs. M1**	-3.833	0.568	-12.089	4.422
	**M0 vs. M2**	-1.667	0.935	-9.922	6.589
	**M2 vs. M4**	5.667	0.256	-2.589	13.922
